# From a Preliminary Lecture

**Published:** 1879-05

**Authors:** 


### ARTICLE II.

### *From a Preliminary Lecture before the Class of the Baltimore College of Dental Surgery.*

BY PROF. HODGKIN.

Gentlemen.—A little more than an hour ago, I was in a city more than forty miles away; and, hurrying along at more than as many miles an hour, I stand before you this evening to give the first lecture of the preliminary course. The fact that I hurry here on the wings or wheels of steam, suggests a theme which may not be unprofitable to think about and talk about for a little while. These are times of swiftness and dispatch. Hurried into the world, hurried through and out of the world, pressed and urged to the utmost tension of mind and body, with all the faculties on the stretch, the American is necessarily immature and unripe. This seems not to be so in other countries and among other nations, and it is not one of the least of our misfortunes that it is so with us. We lack finish, culture, precision, because of the lack of time to be thorough; and this has become constitutional and characteristic of us as a nation. But while this is all true, and while it is to our disadvantage, yet as we are here, and as this is the way all do, we must fall in with the current and, joining the common stream, hurry along to keep up with the eager crowd. You are here to study a profession, one to which hereafter the best efforts of your lives are to be given, and to the acquirement of which the best energies of your brains and fingers are to be devoted. It should, indeed will, take many years to acquire proficiency in this your chosen calling, for proficiency is the child of experience. It ought to be so that several years of patient study could be bestowed here in the lecture room, and in the infirmary and laboratory, on these important themes; but as I have intimated, this is contrary to the genius of our American type of man, and we find that the profession of medicine, and its offshoot dentistry, are mastered, or supposed to be, by the average student in two courses of lectures. I need not tell you that superficiality must result. Still we must do the best we can, earnestly endeavoring to let no opportunity slip of grasping and holding the truths we hear and see.

The array of studies here presented, to a novice, is startling, bewildering and, indeed discouraging. Anatomy, physiology, chemistry, surgical and mechanical dentistry, etc., to be studied and mastered, in outline at least. And as this passes prospectively before you, you are apt to feel as I do, that two seasons are far too little for the attainment of even the outline of this curriculum. But with brave hearts and resolute purposes, and the use of the patient "plodding habit" which is more useful than talent—indeed it *is* a talent —we will accomplish much. There are some here now to whom the words may seem trite, because they have heard them before, but they cannot be too frequently repeated, (and I do it as much for my own application as for yours,) that the genius most to be desired and cultivated, most to be earnestly developed, is a "genius for *hard work*;" the power of steady, continuous application, the ability to not give up. Acquire this habit, united to honest purpose, and success is yours.

Now, I remember as a student, one of the most perplexing things to me was the great prolixity of both lectures and text books. It seemed to me that I should never be able to read all the books, much less master their contents, and as for the lectures, to remember all that was said, seemed simply impossible. By day and by night I toiled, hoping to acquire in detail the works of Harris, Tomes, Gray, Dalton and others, and straining attention over lectures which seemed to be the sum of all knowledge, until utterly discouraged. After a while I learned this thing about studies: The book, the lecture, is a reflection only of the mind of the author, and in his mind are certain truths, facts, theories, he is picturing before his readers or hearers. He has his central idea, his principal theme, his fundamental proposition, and around that he groups his subordinate and correlated facts and arguments. Necessarily there is much outgrowth, important, but subordinate, related to the central fact, but dependent upon it; truths involved in truths. Take this away and the heart of the subject still remains; remove this and the auxiliary facts are irrelevant and useless. So I learned little by little, as years ago I puzzled over the things which now puzzle you, to attack the books and the talk of the lecturer somewhat after this fashion: What is the central fact here? What is this man driving at? What is the pivotal truth of this sentence or page? I learned that just as the farmer cannot grow wheat without also raising straw and chaff, so thought cannot be put down in words without at least something extraneous and subsidiary; not incidental, but necessary indeed to the growth and expression of the thought, just as the straw is necessary to the growth of the grain. Neither thought nor wheat can be produced without these processes, or vehicles, so to speak.

So now, in your studies here, if you can only train your minds to this course—to pounce down upon these precious grains of truth amid their surroundings and treasure them; if you can only learn to scan quickly the sentence and store the pith and marrow of it in your minds, you will have gained a great victory in the outstart. Most of you think you need good memories, and deplore what you call the treachery of that mental power. But I forewarn you that what you need is not so much the powerful and ready memory which reproduces exactly the words of the thinker, the text of the recorder, as the power of swift and accurate generalization ; the power of making an analysis of the *thought* of the study, rather than a recollection of its terms. And understand too (and I hope you will pardon me for opining that you are "green wood" in all things,) that words are only the clothes in which thoughts are dressed, and that ideas are expressed more fully and accurately and precisely by almost any other sign than words. We all realize this unconsciously when we see lectures demonstrated, and say: "I now see perfectly how it is, though I got an idea from the lecturer." This piece of work I hold in my hand, how beautiful, how perfect, how full of suggestion; yet it is silent. I had almost said words are the most lame of our methods of expression. Still we cannot dispense with them ; and you will hear a great deal of talking this winter.

To return : we are here, anxious to learn, full of enthusiasm for our newly adopted profession, intensely engaged in the pursuit of knowledge. None of us, as students, feel fully equipped for the work, and most of us are lamentably deficient, not only in the habit of methodical study, but in the preliminary preparation which should preceed the study of a specialty. Only long and severe training will enable us to compel good habits of mind, and train the thoughts to be obedient servants. So then I advise not so much memorizing as the habit of deduction ; not so much the acquirement of verbiage, as of the conclusions of the speaker or writer. And above all the acquirement of the *thinking faculty.* I had almost rather have you know something of your *own*, than to reply to my questions with re echoes of my voice, rather be made aware that you are *thinking for yourselves*, than recalling what I say. For the very thing you come here for, the "education," is only a "drawing out" of the mind, not a state of passive receptivity. You are not here that I may pour into you, as into a vessel, information, but to respond to thought with thought, to ideas with ideas. And when I ask you questions from week to week, as I shall do this winter, you cannot please me so much as by showing me that you have ideas of your own: you cannot delight me more than by evidencing that you have used your brains.

This pressure which is upon us, as I have intimated, tends to make us superficial. We "skim" over a subject, get a "smattering" of it only, at least this is the tendency. But there is a difference between hurry and dispatch: we may put things through with energy and force, and still not hurry. Hurry is immature; dispatch is mature; the one completes nothing, the other is thorough. Dispatch is all attention, and is able to pass from one subject to another, and mass and concentrate all the energies of mind on what is before it with precision and at once. Indeed, if there be any one great distinguishing mark of a trained mind, it consists in this ability to be able to throw the whole energies on a subject, and at once to pass from that subject to another with facility and completely. But such power comes only by severe training.

And now with our winter's work all before us, you with as yet untried energies, but full of hope; we with what of knowledge and experience the years have brought us,—all with a resolute will that we will accomplish in the hours allowed us, all the work possible, and learn how to *think* as well as how to manipulate,—I think this course of lectures will be all that I desire, and all that you have a right to expect.

One word more: You have the right to think for yourselves, and what I have said so far will show you how pleased I shall be if you do think diligently and accurately; but if you will only take it for granted that the lecturer is the organ, so to speak, of the profession, and that he comes to you fresh from communion with the thinking minds of the age; that what he speaks is as near the truth as it is likely you or most men can get, it will save much trouble. Querulous criticism is useless, and to petulantly find fault with the enunciations of this or that lecturer because his utterances clash with preconceived teachings or ideas, is weakness, not strength, with you. Take it for granted that your lecturer knows his subject, fall in fully with him, and you will be saved much of worry, I may say unhappiness.

